# Detection of Genetic Variants Associated with Behavioural Response During Milking in Simmental Dual-Purpose Cows

**DOI:** 10.3390/ani15121766

**Published:** 2025-06-15

**Authors:** Madalina Mincu-Iorga, Alexandru Eugeniu Mizeranschi, Dinu Gavojdian, Ioana Nicolae, Szilvia Kusza, Daniela Elena Ilie

**Affiliations:** 1 Research and Development Institute for Bovine, 077015 Balotesti, Romania; gavojdian_dinu@animalsci-tm.ro (D.G.); ioana_nicolae2002@yahoo.com (I.N.); 2 Research and Development Station for Bovine Arad, 310059 Arad, Romania; danailie@yahoo.com; 3 Institute for Advanced Environmental Research, West University of Timisoara, 300086 Timisoara, Romania; 4 Faculty of Bioengineering of Animal Resources, University of Life Sciences “King Mihai I” from Timisoara, 300645 Timisoara, Romania; 5 Centre for Agricultural Genomics and Biotechnology, University of Debrecen, 4032 Debrecen, Hungary; kuszasz@hotmail.com

**Keywords:** animal welfare, genetic markers, milking behaviour, Romanian Spotted

## Abstract

Cattle breeding has traditionally focused on improving production traits; however, recent interest in positive animal welfare has shifted attention toward selecting for more robust animals that balance productivity with health and well-being. The aim of the current study was to assess whether behavioural responses during milking in dual-purpose cattle are associated with genetic markers, previously linked to temperament traits in dairy and beef breeds. We focused on 185 lactating cows belonging to the Simmental strain (Romanian Spotted, national name), which were evaluated for their milking behaviour. Genotyping was performed using an 88-SNP panel selected based on prior associations with dairy and beef cattle temperament. We identified five SNPs that were significantly associated with milking reactivity in the Romanian Spotted breed, located in genes previously linked to neural development, stress response and behavioural regulation (*USH2A, ADAMTS7, TBC1D2B* and *ZMAT4*). Our findings suggest that milking behaviour in dual-purpose Simmental cattle is influenced by genetics, supporting the potential for including behavioural traits in future selection strategies. This study contributes to a better understanding of the genetic mechanisms underlying stress-related behaviours in dual-purpose cattle breeds.

## Simple Summary

Cows’ behaviour during milking is an important trait for both their well-being and overall farm efficiency. Calmer cows can make the milking process easier, reduce duration, and increase farm productivity. However, even when proper handling is practiced, some cows remain restless and threaten the safety of their herd mates and that of the animal caretakers. In this study, we investigated whether these behavioural differences are linked to cows’ genetic makeup. The study focused on the Simmental dual-purpose breed, with cows being observed for their behaviour during milking. We identified specific genetic markers that were associated with how cows reacted during milking. The results suggest that behaviour during milking has a genetic component in dual-purpose breeds and that it might be possible to select cows not only for classical traits such as milk yield, but also for workability traits.

## 1. Introduction

Domesticated over 8000–10,000 years ago, cattle (*Bos taurus*) have undergone extensive genetic selection under human management, evolving from animals initially selected for docility to being selected in recent decades primarily for production efficiency [1]. Traditionally, animal breeding shifted towards improving production traits such as milk yield, growth rates and carcass attributes, but recent concerns about the ‘undesirable’ side effects of intensive selection, particularly its impact on animal welfare, have led to a change towards breeding for more robust and resilient animals, which prioritizes health and welfare alongside productivity [2,3,4].

As a wide range of studies suggest, human–animal interaction significantly affects the behaviour [5], physiology [6] and productivity [7] of cattle raised for both dairy and beef. One common approach used to assess cattle responsiveness to humans is to measure their behaviour during milking [8,9,10,11]. Milking behaviour, often assessed through actions such as kicking or stepping during milking [12], reflects immediate, context-specific responses to the milking process, rather than stable behavioural traits. In contrast, temperament refers to consistent behaviour across multiple situations [13]. Previous studies have shown that a single observation of milking behaviour is often sufficient, due to its temporal stability [8,14]. Therefore, we refer to this trait as “milking behaviour” or “behaviour during milking” throughout this article, to distinguish it from the broader concept of temperament.

Individual variation among cows in coping with stress and environmental challenges affects milk performance, milk ejection rates, fertility, health, metabolic efficiency, enteric CH_4_ emissions and animal welfare [15,16,17,18,19,20,21], with the studies being implemented almost exclusively on Holstein-Friesian breeds. Dual-purpose breeds may offer greater robustness and adaptability in variable environments, which could support their relevance for behavioural studies in diverse production systems [22]. With an estimated census of over 40 million animals, the Simmental breed represents one of the most widely distributed cattle breeds world-wide [23,24]. Furthermore, calmer dairy cows tend to have lower culling rates, likely because farmers are more inclined to cull animals that are difficult to milk [17]. In beef cattle, calmer animals grow at faster rates [25] and demonstrate better feed conversion efficiency [26]. Moreover, their meat quality is often superior [27,28] and they show improved health and reproduction efficiency [29].

The rapid expansion of large-scale genetic variant databases has emphasized the importance of identifying genes or loci responsible for controlling phenotypic traits [30]. While it is generally acknowledged that genetic variations influence cattle behaviour, our understanding of how genes translate into specific behavioural traits remains limited. One reason is the complexity of the behavioural traits, which are typically influenced by multiple genetic loci [31]. Single nucleotide polymorphism (SNP) arrays have emerged as the primary genotyping technology used in genome association studies in cattle [32,33] due to their affordability and time-efficiency, making them particularly suitable for use due to the relatively low costs [34]. Recent studies using SNPs in dairy and beef cattle identified genetic variations that are associated with behavioural traits [35,36,37], with limited research being conducted on dual-purpose breeds to date.

The aim of this study was to investigate whether behavioural response during milking is associated with specific SNPs in Romanian Spotted (Simmental strain) dual-purpose cattle breed.

## 2. Materials and Methods

### 2.1. Animals’ Management and Behaviour Assessment

The study was carried out from August to September 2022 at the Research and Development Station for Bovine Arad, Romania, on 185 lactating Romanian Spotted (Simmental strain) dual-purpose cows between their 1st and 3rd lactations. The animals’ age at calving had an average of 1681 ± 217 days.

All cows were purebreds and were included in the performance and recording scheme as well as in the herd-book, with Simmental genetics from Austria, Switzerland and Germany being introduced and routinely used throughout semen imports and practicing artificial insemination.

Cows were milked twice per day in a ‘herringbone’ (2 × 14 units) milking parlour (starting at 5:00 and 17:00). Animals were equipped with AfiTag^®^ pedometers (Afimilk Ltd., Kibbutz Afikim, Israel) and monitored for milk yield, milking speed and milk conductivity using AfiFarm 5.4^®^ farm management software. Cows were housed in a loose system, kept on deep straw bedding, with a space allowance of 9 m^2^/animal in the resting area and free access to outside paddocks. Cows were fed twice per day having ad libitum access to water, and were housed in groups up to 50 animals with a feeding space allowance of about 75 cm/head. The daily ration consisted of 6 kg alfalfa hay, 6 kg concentrates and 35 kg maize silage.

Cow’s milking behaviour was evaluated once using a 5-point scoring system [8], using one trained observer placed in the parlour during milking, cows being scored as follows: Score 1: the cow is ruminating, calm and relaxed, with no additional movements; Score 2: the cow is alert but calm, with occasional head and ear movements, keeping the head forward; Score 3: the cow is alert and reactive to the milking machine being attached and removed, with moderate movements, bouncing the head back and forth; Score 4: the cow is kicking and shifting her weight from one hind leg to the other, defecating or urinating, and exhibiting abrupt, episodic movements. Finally, a cow scoring 5 kicks and attempting to remove the milking machine, appears obviously restless, vocalizes and defecates or urinates, with continuous episodic movements. Although intraobserver reliability was not calculated, the observer underwent 3 months of training with another experienced observer. During this period, both observers independently scored the same animals during milking and their scores were compared at the end of milking to ensure consistency.

Body condition score (BCS) was recorded once for each cow on the same day as the milking reactivity assessment using the WelfareQuality^®^ protocol [38] scale for dual-purpose cows, with score 0 indicating a regular body condition, score 1 representing very lean cows and score 2 denoting very fat/obese cows. Udder cleanliness (0—no dirt or minor splashing; 2—separate or continuous plaques of dirt), tarsal joint lesions (0—presence/2—absence) and claw overgrowth as a lameness indicator (0—no overgrowth; 2—medium or severe overgrowth) were recorded as binomial variables.

Milk yield and quality data were taken from the results of one official performance recording, according to the standardized International Committee for Animal Recording (ICAR) guidelines [39], specifically on 30 August 2022, with cows being balanced for days in milk (DIM). Milk yield per test day (kg), milk fat percentage per test day (%) and milk protein percentage per test day (%) were recorded individually, alongside the somatic cell score (SCS) which was calculated using the following formula: SCS = log_2_ (SCC/100,000) + 3, in order to normalize its distribution and reduce skewness in statistical analyses. In the formula, SCC = Somatic Cell Count (cells per millilitre of milk). Although milking speed is a relevant trait commonly included in behavioural studies, it was not evaluated in the present study due to malfunctioning milk meters in the milking parlour during the observation period, which prevented reliable data collection.

To investigate the association between behaviour during milking and performance or health indicators in dual-purpose cows, separate linear regression analyses were conducted for each of the following outcome variables: milk yield (kg/day), milk fat percentage (%), milk protein percentage (%) and somatic cell score (log-transformed cells/mL). Each regression included the following fixed effects: parity, days in milk (DIM), milking behaviour score, body condition score (BCS), udder cleanliness, tarsal joint lesions and claw overgrowth. While multiple explanatory variables were included to control for potential confounders, the present analysis focuses specifically on the effects associated with milking behaviour.

### 2.2. Selection of the SNPs Panel Related to Milking Behaviour in Cows

We conducted a stepwise gene selection process, starting with the identification of candidate genes, followed by the selection of relevant single nucleotide polymorphisms (SNPs). Firstly, we screened a recent comprehensive review [40] that examined genetic associations with bovine behaviour traits, including traits such as ‘temperament’, ‘docility’, ‘aggression’, ‘flight speed’, ‘chute score’ and ‘milking temperament’. Secondly, we conducted an association study with the aim of replicating some previously identified behavioural genetic associations and assess their relevance in our cattle population which has a different genetic architecture.

A total of 88 SNPs were chosen across the cattle genome, based on previous results from the literature review. The selected SNPs belong to 24 genes associated with ‘milking temperament’: *GAP43* (Growth-Associated Protein 43), *KCNJ3* (Potassium Inwardly Rectifying Channel Subfamily J Member 3), *CD2* (Cluster of Differentiation 2), *IGSF3* (Immunoglobulin Superfamily Member 3), *NXNL2* (Nucleoredoxin Like 2), *GRIN3A* (Glutamate Ionotropic Receptor NMDA Type Subunit 3A), *TBC1D32* (TBC1 Domain Family Member 32), *RARS2* (Arginyl-tRNA Synthetase 2, Mitochondrial), *RPS6KA2* (Ribosomal Protein S6 Kinase A2), *NEO1* (Neogenin 1), *HCN4* (Hyperpolarization-Activated Cyclic Nucleotide-Gated Channel 4), *KCTD3* (Potassium Channel Tetramerization Domain Containing 3), *USH2A* (Usherin), *HSPB8* (Heat Shock Protein Family B Member 8), *SRRM4* (Serine/Arginine Repetitive Matrix 4), *OTUD7A* (OTU Deubiquitinase 7A), *ADAMTS7* (A Disintegrin And Metalloproteinase With Thrombospondin Motifs 7), *TBC1D2B* (TBC1 Domain Family Member 2B), *EEFSEC* (Eukaryotic Elongation Factor, Selenocysteine-tRNA-Specific), *RUVBL1* (RuvB Like AAA ATPase 1), *KBTBD12* (Kelch Repeat and BTB Domain Containing 12), *ZMAT4* (Zinc Finger, Matrin-Type 4); ‘flight speed’: *BTC* (Betacellulin); ‘temperament and habituation’: *ADCY2* (Adenylate Cyclase 2). Genes were included based on their previously reported associations with behavioural traits in cattle and their involvement in neurobiological processes such as sensory perception, synaptic function and stress response regulation.

The SNPs for the investigated candidate genes were selected based on the microarray Axiom Bovine Genotyping v3 Array (Thermo Fisher Scientific, Waltham, MA, USA), which contains over 63,000 SNP markers. This SNP array includes approximately 44,000 SNPs from the Council on Dairy Cattle Breeding (CDCB), core parentage markers recommended by the International Society for Animal Genetics (ISAG), SNPs linked to key traits of interest, as well as markers selected for improved imputation of short tandem repeats (STRs). The final list of SNPs used in the association study including the details regarding the Affy SNP ID/rs ID, gene symbol, chromosome position based on the reference genome Bos_taurus_UMD_3.1.1, flanking region and alleles are provided in Supplementary file S1.

### 2.3. Sampling and Genotyping

The procedures and sample collection protocols were previously approved by the Institutional Ethics Committee of the Research and Development Station for Bovine Arad, Academy for Agricultural and Forestry Sciences (Decision no. 88/4 October 2019). Blood samples were collected by trained veterinarians. The genotyping workflow and data processing were carried out as described previously by Ilie et al. [41]. Briefly, genotyping was performed using the Axiom_BovMDv3 SNP array (Thermo Fisher Scientific). Any SNPs with a call rate below 95% were excluded from further analysis and SNPs with genotypes not in accordance with the Hardy–Weinberg equilibrium (*p* > 106) were eliminated. Individuals with a call rate of less than 95% were also excluded. The current SNP data-set represents a sub-set of the genotyping data used in the work previously published by Ilie et al. [41], which comprised 601 cattle.

The research activities were performed in accordance with the European Union’s Directive for animal experimentation [42], with the milking behaviour assessment causing no pain nor distress to the cows.

### 2.4. Statistical Analysis

Various statistical analyses were carried out using the R programming language v. 4.3.3 (R Core Team) [43].

Descriptive statistics were collected using the psych package v. 2.4.3 [44], and a principal component analysis on genotypes was carried out using the argyle package v. 0.2.2 [45]. Results were visualized using the packages corrgram v. 1.14 [46] and ggplot2 v. 3.5.1 [47]. Linear regression analyses were performed using R’s built-in *lm* and *glm* functions. The latter was used for Poisson-distributed phenotypes, with the option *family = poisson (link =* “*log*”*)*.

For genotype association, genotypes were represented as ordinal categorical variables (AA < AB < BB for 2 alleles A and B), and the *clm* function from the R package ordinal v. 2023.12-4.1 was used [48], with the first 6 principal components included as covariates in order to account for population stratification. This implements a proportional-odds cumulative link model with the following formulation for an individual i with the following covariance vector:

$$x_{i}=({SNP}_{i},\;{PC1}_{i},\ldots ,{PC6}_{i})$$

and the phenotype vector $Y_{i}\in \{1,\ldots ,J\}$ denoting the ordered categories of milking behaviour. The proportional-odds cumulative link model fitted by the *clm* function is

$$F(P(Y_{i}\leq j))=\theta_{j}-x_{i}^{⊤}\beta ,\;j=1,\ldots ,J-1,$$

where

F is the link function, which by default is the logit function: $logit(p)=log(\frac{p}{1-p})$;
j is the category of $Y_{i}$;
$P(Y_{i}\leq j|x_{i})$ is the cumulative probability of being in category j or below;
$\theta_{j}$ are threshold parameters (intercepts);
β is the vector of regression coefficients.

Expanding the previous equation and using the logit link function gives the following form:

$$log\left(\frac{P(Y_{i}\leq j)}{P(Y_{i}>j)}\right)=\theta_{j}-x_{i}^{⊤}\beta$$

or, conversely,

$$log\left(\frac{P(Y_{i}>j)}{P(Y_{i}\leq j)}\right)=x_{i}^{⊤}\beta -\theta_{j}$$

The *clm* R function estimates the regression coefficients via maximum likelihood estimation. In the case of SNP effects $\beta_{SNP}$, they can be interpreted as the log-odds ratio of being in a higher versus lower category j for the phenotype Y (milking behaviour), for each additional copy of the B allele. Positive $\beta_{SNP}$ values depict that the presence of additional B alleles is associated with higher milking reactivity categories, while the opposite relationship is inferred for negative $\beta_{SNP}$ values. Exponentiating the $\beta_{SNP}$ coefficients yields the corresponding odds ratios (OR). Positive $\beta_{SNP}$ coefficients have corresponding OR > 1, while negative coefficients yield OR < 1.

For the regression models mentioned above, pairwise Tukey comparisons of each pair of genotypes were implemented with the R package emmeans v. 1.10.6, Bonferroni correction was applied to the *p*-values, and the results were considered statistically significant at a threshold of *p* ≤ 0.05.

## 3. Results

### 3.1. Phenotypic Data

A non-linear relationship was observed between behaviour during milking and milk yield, with the negative fourth-order term indicating that higher reactivity levels were associated with lower milk production. Milking behaviour was modelled as an ordered factor ranging from 1 to 5, with linear (estimate = 0.18; *p* = 0.556), quadratic (estimate = −0.29; *p* = 0.325), cubic (estimate = 0.06; *p* = 0.839) and quartic (estimate = −0.72; *p* = 0.007) coefficient estimates, the latter being statistically significant. The model explained approximately 23% of the variation in milk yield (R^2^ = 0.2286; adjusted R^2^ = 0.1987), with an overall model significance of F = 6.54; *p* < 0.001.

Within the model used for milk fat percentage analysis, milking behaviour did not have a significant effect, as none of the polynomial terms reached statistical significance (linear: estimate = 0.11; *p* = 0.639, quadratic: estimate = −0.01; *p* = 0.974, cubic: estimate = −0.04; *p* = 0.816, quartic: estimate = 0.16; *p* = 0.448). The model explained approximately 14% of the variation in fat percentage (R^2^ = 0.130; adjusted R^2^ = 0.091), while the overall model was statistically significant (F = 2.92; *p* = 0.0009).

In the milk protein percentage model, milking behaviour was included as a predictor using a polynomial structure. The linear term (estimate = −0.108; *p* = 0.083) was not statistically significant but suggested a potential downward tendency. The quadratic (estimate = 0.005; *p* = 0.976), cubic (estimate = −0.031; *p* = 0.872), and quartic (estimate = −0.036; *p* = 0.857) terms were also not statistically significant. The model explained approximately 35% of the variation in protein percentage (R^2^ = 0.347; adjusted R^2^ = 0.3093), with an overall model significance of F = 9.621; *p* < 0.001.

In the model for the somatic cell score (SCS), the quartic term for milking behaviour was statistically significant (estimate = −0.7214; *p* = 0.007), suggesting a complex non-linear relationship. The linear (estimate = 0.005; *p* = 0.982), quadratic (estimate = 0.004; *p* = 0.985) and cubic (estimate = −0.036; *p* = 0.881) contrasts were not significant (*p* > 0.05), implying that SCS did not show a straightforward association with reactivity levels. The model explained approximately 23% of the variation in SCS (R^2^ = 0.2235; adjusted R^2^ = 0.1906), with overall model significance (F = 6.203; *p* < 0.001). Detailed model coefficients and significance values are provided in Supplementary file S2.

### 3.2. Genotypic Data

We identified 5 SNPs significantly associated with behavioural response during milking in Romanian Spotted (Simmental strain) cows. The allele and genotype frequency distributions for these SNPs are presented in Table 1, while Table 2 provides details on their genomic locations. SNP effect sizes are expressed as log-odds ratios between groups for corresponding genotypes.

Among the identified SNPs, rs41609061 in *USH2A* (AX-106757017, chromosome 16) displayed three genotype frequencies, AA (26%), AG (56%) and GG (18%), with allele frequencies of A = 54% and G = 46%. Cows with the AG genotype exhibited lower milking behaviour scores compared to AA homozygotes, as reflected by a negative SNP effect size (−1.15), suggesting that *USH2A* gene may influence sensory perception and responsiveness during milking.

The rs110435789 in *ADAMTS7* gene (AX-106728113, chromosome 21) showed genotype distributions of CC (9%), CT (30%) and TT (61%), with C = 23.5% and T = 75.5% allele frequencies. The CC genotype was associated with a stronger behavioural response during milking compared to CT heterozygotes, as indicated by a positive SNP effect size (2.10). This suggests a potential role of *ADAMTS7* in milking behaviour, a gene involved in extracellular matrix remodelling and motor control.

Two significant SNPs were detected within the *TBC1D2B* gene (rs41960181/AX-106723972 and rs41960216/AX-185115752, on chromosome 21), with identical genotype distributions, AA (65%), AC/AG (27%) and CC/GG (8%), with allele frequencies of A = 77.5% and C/G = 21.5%. Cows with the CC genotype for rs41960181 and GG genotype for rs41960216 displayed higher scores for milking behaviour compared to heterozygotes, with a positive SNP effect size of 2.05 for both SNPs. The presence of multiple significant SNPs within *TBC1D2B* suggests that this gene may play an essential role in intracellular signalling pathways related to stress responses during milking.

Finally, the *ZMAT4* gene (rs110175723/AX-124348710, chromosome 27) exhibited a predominant GG genotype (90%), with AG heterozygotes accounting for 10% and no observed AA individuals. The G allele frequency was 95%, indicating a high degree of fixation in this population. The AG genotype was associated with lower reactivity scores than GG homozygotes, as demonstrated by a negative SNP effect size (−1.52). This suggests that *ZMAT4*, a zinc finger protein, may have a regulatory function in neurodevelopmental processes affecting behavioural responses in dual-purpose cows. Bar plots showing genotype and allele frequencies for all significant SNP markers are presented in Supplementary file S3. Detailed contrasts and *p*-values associated with SNP effects on milk production traits are provided in Supplementary file S4.

## 4. Discussion

To better understand the genetic basis of milking behaviour, the functional background and behavioural implications of the five associated genes identified in our study are presented below. The *USH2A* gene is considered to play a role in pre- and postsynaptic membrane adhesion as well as nerve fibre guidance, mainly in the basement membrane of the cochlea (inner ear) and retina. Mutations in *USH2A* are responsible for a subtype of Usher syndrome, the most common cause of combined deaf-blindness in humans [49]. In animals, the *USH2A* gene has been linked to the nervous metabolic system [50]. The *USH2A* gene, located on chromosome 16, was identified as a candidate gene associated with milking temperament in a study involving Canadian Holstein dairy cows. It has been previously reported a significant SNP within the 3′ downstream region of *USH2A* gene on *Bos taurus* chromosome 16 (BTA16), alongside the potassium channel tetramerization domain containing 3 (*KCTD3*) gene, suggesting a potential role in behavioural traits related to milkability [34]. Given its role in sensory processing, USH2A may influence how cows perceive tactile or auditory stimuli during milking, potentially modulating their behavioural response. We found that in the case of SNP rs41609061, located in *USH2A* gene, the AG genotype exhibited lower milking behaviour scores compared to AA homozygotes.

The *ADAMTS7* gene, also referred to as COMP-ADAMTSs due to its role in degrading cartilage oligomeric matrix protein (COMP), has been found to be significantly elevated in conditions such as osteoarthritis and rheumatoid arthritis [51]. Additionally, its expression is markedly upregulated in cardiomyopathies, arterial ruptures, neointimal transformation and atherosclerotic calcification [52]. Our results show that the CC genotype of SNP rs110435789 in the ADAMTS7 gene is associated with a stronger behavioural response during milking. Its involvement in tissue remodelling could influence muscle tone or joint sensitivity, which may in turn affect reactivity during udder manipulation.

While specific functional studies on *TBC1D2B* in cattle are limited, its role in intracellular trafficking suggests potential implications for various physiological processes. Moreover, *TBC1D2B* has been identified as a candidate gene involved in the regulation of growth and weight gain in Red Brown Korean native chickens [53]. In our study, the two SNPs located on the *TBC1D2B* gene (rs41960181 and rs41960216) played an important role in the behavioural response during milking, with the CC and GG genotypes, respectively, displaying higher scores for milking reactivity compared to heterozygotes. We speculate that TBC1D2B may play a role in how cows process stress-related stimuli during milking, through intracellular signalling mechanisms.

In animals, the *ZMAT4* gene is involved in apoptotic, biological, developmental and metabolic processes [54]. Previous studies reported that *ZMAT4* gene is associated with milk yield at the beginning of lactation, milking speed and temperament in dairy cattle [34,55]. In Holstein dairy cattle, it has been linked to quantitative trait loci (QTLs) associated with dystocia [56] and calving ease [57], while in Angus beef cattle, it has been associated with the non-return rate [58]. Our results highlight that the variations in rs110175723 located on the *ZMAT4* gene are associated with lower reactivity scores in the case of the AG genotype. As a transcriptional regulator, ZMAT4 may influence neurodevelopmental processes underlying behavioural responses to routine handling. However, given the high degree of fixation for this gene’s allele frequencies in our study, the generalizability of our results to other Simmental populations will need to be established in future studies.

These gene-level insights lay the groundwork for exploring how milking behaviour can be integrated into future genetic improvement strategies. Behaviour during milking in dairy cattle is generally regarded as having a low to moderate heritability, with estimates ranging from 0.07 to 0.13 [17,20,59], thus making it a candidate trait with relatively low expected genetic gains for selection. However, given the genetic correlations of reactivity with milk production, health and reproductive traits, and the newly established associations of behavioural traits with the SNPs found on panels already used for cattle selection, the five SNPs described above could be included in future selection schemes for the Simmental group breeds. Although milking reactivity exhibits low to moderate heritability, the identification of associated SNPs offers the potential to improve selection accuracy, particularly when combined with other indicators in multi-trait genomic prediction models. Even modest gains may be relevant for traits that also affect animal welfare and farm safety. Furthermore, Sewalem et al. [8], working with an impressive database of over 1,940,000 records on Holstein cattle, outlined that behaviour during milking could be assessed just once, given the consistency of the trait during the cattle’s productive life. Moreover, milking behaviour could be relatively easy to assess during regular on-farm milking recordings, is inexpensive and the assessment methods could be standardized.

Our findings have applied relevance to the management and selection of commercial dual-purpose cattle. Previous work by Cziszter et al. [11] had shown that milking behaviour has a significant influence on body weight, milk yield, milk ejection speed, fat yield, protein yield, calving interval and the number of steps per day of Simmental cows, with calmer cows outperforming their nervous counterparts, the research being undertaken at the same research station as that of the current study. Our results provide a genetic complement to the phenotypic observations reported by Cziszter et al. [11], reinforcing the notion that calmer cows perform better across multiple traits. The SNPs identified here may contribute to the underlying genetic basis of those performance differences, supporting their relevance in selection strategies for dual-purpose breeds. Improving behavioural traits such as milking behaviour may also contribute indirectly to better longevity in Simmental cows, as recent research has shown that functional traits like body condition score and muscularity significantly influence stayability in dual-purpose breeds [60].

Given that milking behaviour trait is consistent from the first lactation to further lactations, there is the potential to identify primiparous cows that are most likely to do well or poorly when faced with stressful farm management practices, and thus take into account such factors for selection decisions. Even in calmer cows, physiological events such as calving may transiently influence behavioural responses, as shown by Mammi et al. [61]. This underlines the importance of considering both genetic predisposition and life-stage context when evaluating behaviour. Additionally, we acknowledge that efforts to improve the dissemination of behavioural genetics findings among dairy producers, whether through tailored outreach or professional platforms could enhance knowledge transfer and increase the overall impact of our findings. For instance, Lamanna et al. [62] explored how social media, may support applied veterinary education by fostering engagement and knowledge uptake in dairy nutrition.

## 5. Conclusions

We identified five SNPs that are significantly associated with milking behaviour in Romanian Spotted cows. Several of these variants fall within genes previously linked to neural regulation, stress response and behavioural traits in Holstein dairy cattle, suggesting a genetic contribution to how cows respond to the milking procedure.

Our findings support the idea that milking behaviour might be a biologically relevant trait with implications for both productivity and animal welfare. As a result, including behavioural indicators such as reactivity in genomic breeding strategies could contribute to the selection of animals that cope better with their environments, ultimately leading to improved farm efficiency and better welfare outcomes. However, these findings should be interpreted with caution, as the study was conducted in a single population and the effects of individual SNPs on complex behavioural traits are likely to be modest. One limitation of this study is the sample size, which may limit the ability to detect small genetic effects for complex behavioural traits. Additionally, production traits were recorded on a single test day, which may not fully capture their variability over time.

Future studies should aim to integrate genomic and physiological data, such as SNP associations and stress biomarkers, in order to develop more accurate genomic tools for the selection of behavioural traits. Also, further validation in larger and genetically diverse populations is needed to confirm the broader applicability of these associations.

## Figures and Tables

**Table 1:** Allele and genotype frequency distributions for the significant SNPs in the Romanian Spotted (Simmental strain) dual-purpose cows.

SNP ID	Gene	Genotype Frequency (*n*)			Allele Frequency		Genotype Effect	SNP Effect Size
AX-106757017	*USH2A*	AA	AG	GG	A	G		
		0.260 (31)	0.560 (66)	0.180 (21)	0.540	0.460	AG < AA	−1.15
AX-106728113	*ADAMTS7*	CC	CT	TT	C	T		
		0.090 (11)	0.300 (35)	0.610 (72)	0.235	0.765	CC > CT	2.10
AX-106723972	*TBC1D2B*	AA	AC	CC	A	C		
		0.650 (76)	0.270 (32)	0.080 (10)	0.775	0.225	CC > AC	2.05
AX-185115752	*TBC1D2B*	AA	AG	GG	A	G		
		0.650 (76)	0.270 (32)	0.080 (10)	0.775	0.225	GG > AG	2.05
AX-124348710	*ZMAT4*	AA	AG	GG	A	G		
		0.000 (0)	0.100 (12)	0.900 (106)	0.050	0.950	AG < GG	−1.52

**Table 2:** Details of genes, chromosome location and genomic location of significant SNPs found for behavioural response during milking in Romanian Spotted (Simmental strain) cattle.

SNP ID	Gene Symbol	SNP rs ID	Chr	Position ^1^	Alleles	Flanking Region
AX-106757017	*USH2A*	rs41609061	16	20099741	A/G	CAATAAAACAGGACA[A/G]CCTCAAGGGAAGCAT
AX-106728113	*ADAMTS7*	rs110435789	21	30887167	C/T	CCACATCCAGCCGCC[C/T]GAGTTCTCCTGGCGC
AX-106723972	*TBC1D2B*	rs41960181	21	30908358	C/A	CTATGGCCTGAAGCC[A/C]CTCATACACTCCAAA
AX-185115752	*TBC1D2B*	rs41960216	21	30917279	G/A	TCATATTCAAATGGC[A/G]CTGTGATACATGATC
AX-124348710	*ZMAT4*	rs110175723	27	35339647	A/G	ATCACCATCAGCTTT[A/G]ATGGCCATCTAGAAA

^1^ Position based on the UMD3.1 genome assembly of Bos taurus. Chr: chromosome.

## Data Availability

Data will be available upon request.

## Supplementary Materials

The following supporting information can be downloaded at: https://www.mdpi.com/article/10.3390/ani15121766/s1, Supplementary file S1: List of SNPs used in the association study including the details regarding the Affy SNP ID/rs ID, gene symbol, chromosome position based on the reference genome Bos_taurus_UMD_3.1.1, flanking region and alleles [34,63]; Supplementary file S2: Effects of variables of interest on milk traits; Supplementary file S3: Genotype and allele frequencies for all significant SNP markers; Supplementary file S4: SNP effects on milk traits: significant contrasts and *p*-values.
