# Supplementary figures and images for: Detection of Genetic Variants Associated with Behavioural Response During Milking in Simmental Dual-Purpose Cows
Source: Animals (Basel). 2025 Jun 15;15(12):1766. doi: 10.3390/ani15121766 (PMC12189442; doi:10.3390/ani15121766)

Genotype and allele frequencies for all significant SNP markers

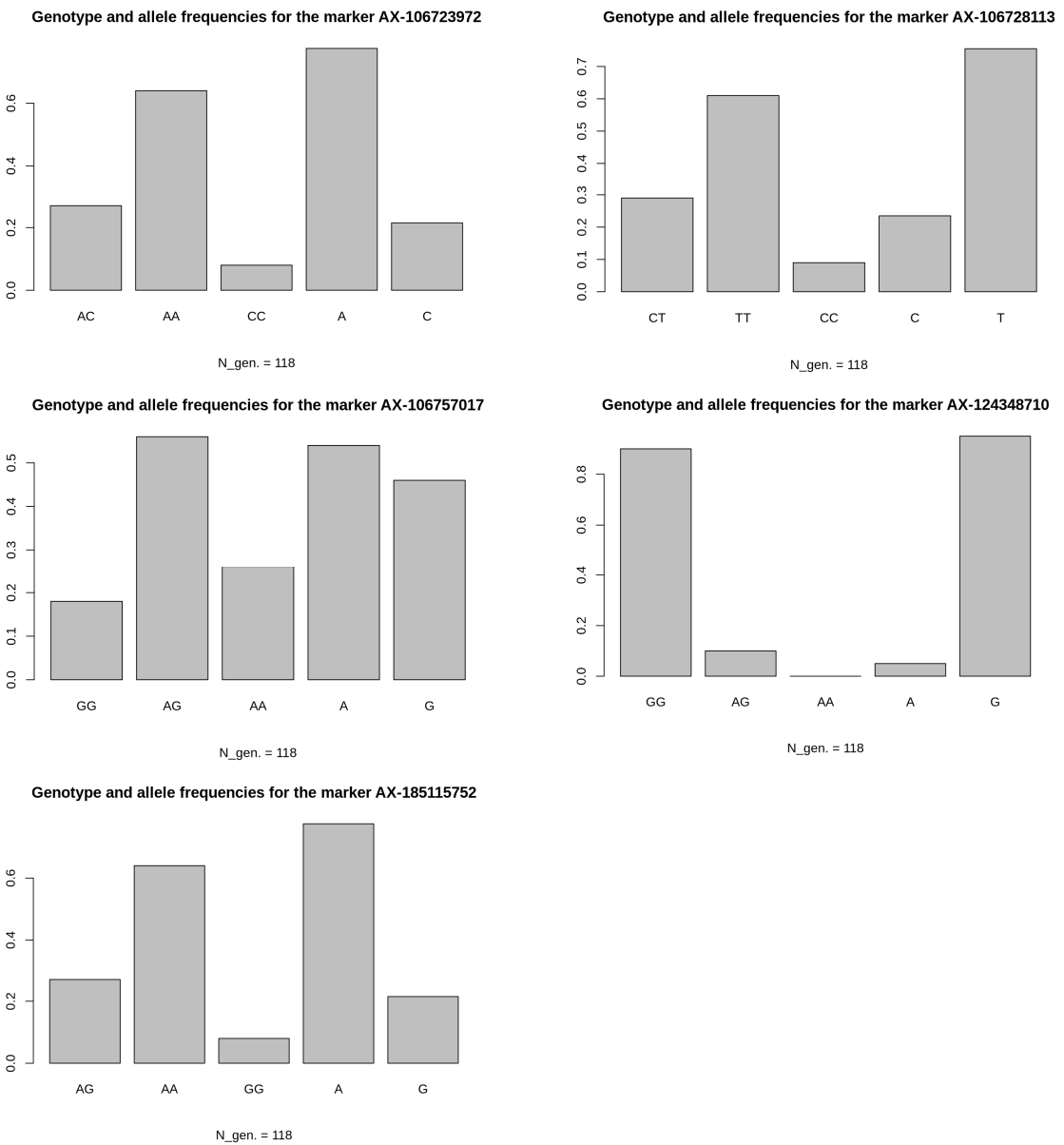

Supplement: Supplementary file 1 [file animals-15-01766-s001.zip › Supplementary file S3.pdf]
